# Fungal Pigments and Their Roles Associated with Human Health

**DOI:** 10.3390/jof6040280

**Published:** 2020-11-12

**Authors:** Lan Lin, Jianping Xu

**Affiliations:** 1 School of Life Science and Technology, Department of Bioengineering, Key Laboratory of Developmental Genes and Human Diseases (MOE), Southeast University, Nanjing 210096, Jiangsu, China; linl04@seu.edu.cn; 2 Department of Biology, McMaster University, Hamilton, ON L8S 4K1, Canada

**Keywords:** fungal pigments, melanin, carotenoids, polyketides, azaphilones, antitumor, medical roles

## Abstract

Fungi can produce myriad secondary metabolites, including pigments. Some of these pigments play a positive role in human welfare while others are detrimental. This paper reviews the types and biosynthesis of fungal pigments, their relevance to human health, including their interactions with host immunity, and recent progresses in their structure–activity relationships. Fungal pigments are grouped into carotenoids, melanin, polyketides, and azaphilones, etc. These pigments are phylogenetically broadly distributed. While the biosynthetic pathways for some fungal pigments are known, the majority remain to be elucidated. Understanding the genes and metabolic pathways involved in fungal pigment synthesis is essential to genetically manipulate the production of both the types and quantities of specific pigments. A variety of fungal pigments have shown wide-spectrum biological activities, including promising pharmacophores/lead molecules to be developed into health-promoting drugs to treat cancers, cardiovascular disorders, infectious diseases, Alzheimer’s diseases, and so on. In addition, the mechanistic elucidation of the interaction of fungal pigments with the host immune system provides valuable clues for fighting fungal infections. The great potential of fungal pigments have opened the avenues for academia and industries ranging from fundamental biology to pharmaceutical development, shedding light on our endeavors for disease prevention and treatment.

## 1. Introduction

Since prehistoric times, fungi have played critical roles in human daily activities, such as baking, brewing (wines and beers), and processing dairy products. With the advent of new technologies, especially biotechnology, over the past 60 years, the use of fungi has increased significantly for the production of commercially important products, such as edible mushrooms, drinks, alcohols, organic acids, enzymes, and colorants. After the breakthrough discovery of penicillin in the 1930s, fungi have become a very significant source of pharmaceutical products which give rise to life-saving medicines, including antibiotics, anticancer drugs, and cholesterol-lowering agents [1]. Apart from the benefits that fungi provide, some fungi can also cause significant diseases to a variety of crops, livestock, pet animals, and humans. Accumulating evidence has revealed that the incidence of human fungal diseases has been increasing rapidly since the 1980s, and is associated with excessive morbidity and mortality, particularly among immunosuppressed and immunocompromised patients [2]. Among the 625 fungal species known to be pathogenic to humans, those in the genera *Aspergillus*, *Candida*, *Cryptococcus*, and *Trichophyton* cause diseases in over 300 million people globally [2]. These diseases can be superficial, subcutaneous, or systemic. Some are acute infections, but others can be long-lasting chronic infections (e.g., athlete’s foot). Accordingly, the detailed biological processes for generating useful products from beneficial fungi and for virulence in pathogenic fungi are very active areas of research.

To the general public, when we talk about pigments in living organisms, we often think of the colorful flowers, insects, and birds. Indeed, pigments in these groups of organisms are the subjects of curiosity for people from diverse walks of life, including painters, photographers, writers, gardeners, and biologists. The biological roles for many of those pigments have been extensively studied. In contrast, unless we are in the woods and see colorful mushrooms, we often do not associate pigments with fungi, with the possible exception of “black” molds in spoiled foods. However, fungi are prolific producers of a myriad of pigments of different chemical structures and a diverse range of colors. The majority of well-studied fungal pigments are from fungi of four genera: *Aspergillus*, *Penicillium*, *Paecilomyces,* and *Monascus* [3,4]. When chemically categorized, fungal pigments are grouped into carotenoids, melanins, polyketides, azaphilones (polyketide derivatives), etc. [5,6].

Carotenoids constitute an abundant group of pigments in nature. They are found in all plants where they play an essential role in photosynthesis. In fungi, over 200 fungal species have been documented as capable of producing carotenes [7]. Carotenoids play very important roles in human health. They not only serve as the precursors of vitamin A in humans, but can also alleviate and/or prevent human age-related diseases, such as cataracts and macular degeneration. Furthermore, they can reduce the incidence of coronary heart disease and carcinomas, including lung, breast, prostate, and colorectal cancers [8]. The health-promoting effects of carotenoids are believed to be associated with their bioactivity as antioxidants and free radical scavengers.

Another broadly distributed group of fungal pigments is melanin. Melanin contributes significantly to the fungal capacity to survive and thrive in unfavorable habitats. Fungal melanin molecules are polyphenolic and/or polyindolic compounds with high molecular masses and negative charges. Due to its physiochemical properties, melanin can mediate an array of cellular functions, allowing fungal adaptation to diverse environmental factors, including ultraviolet (UV) light, heat, ionizing radiation, and oxidative stressors [9]. These properties make melanin a potential artificial stress-protecting agent (e.g., radioprotective) in bioinspired applications. However, the relevance of melanin to human health is probably best exemplified in human fungal pathogens as a virulence factor. For example, in the opportunistic human pathogens of the *Cryptococcus* genus, such as *Cryptococcus neoformans* and *C. gattii*, strains unable to produce melanin are avirulent or have significantly reduced virulence [10,11]. Similarly, in *Aspergillus fumigatus* as well as *Paracoccidioides brasiliensis*, the melanized cells exhibited elevated resistance to phagocytosis [12].

In this paper, we provide a comprehensive and updated review on fungal pigments that have demonstrated relevance to human health either directly or indirectly. We first describe the categories and chemical structures of these fungal pigments, including representative fungi that produce them. This is followed by descriptions of their biosynthesis pathways, how they interact with the human immune system, and the relationships between their structure and activities. We finish by providing a perspective on future developments.

## 2. Types of Fungal Pigments and Their Relevance to Human Health

The majority of organisms living on this planet synthesize some pigments. These biological pigments confer on our world a wide array of colors via the absorption and refraction of specific wavelengths of light. In nature there are many types of biological pigments, ranging from monomeric (such as carotenoids, flavonoids, luciferin, and heme/porphyrin-based pigments) to polymeric (i.e., melanin and humic compounds). Some pigments possess conjugated moieties (i.e., aromatics rings) that facilitate electronic resonances and mediate energy transfers within and between cells. The energy captured and/or reflected by pigments has been shown to play multiple biological roles ranging from the maintenance of life, including the utilization of solar energy for metabolic functions and protection against radiation damage, to camouflage, as well as mate and pollinator attraction. Below, we describe the main types of fungal pigments relevant to human health.

### 2.1. Carotenoids

In addition to being produced by photosynthetic organisms ranging from cyanobacteria to flowering plants, carotenoids are also synthesized in a variety of heterotrophic microbes including fungi [13]. Furthermore, carotenoids are present in animals that are unable to synthesize them de novo, but instead acquire them from their diet.

**Figure 1:**
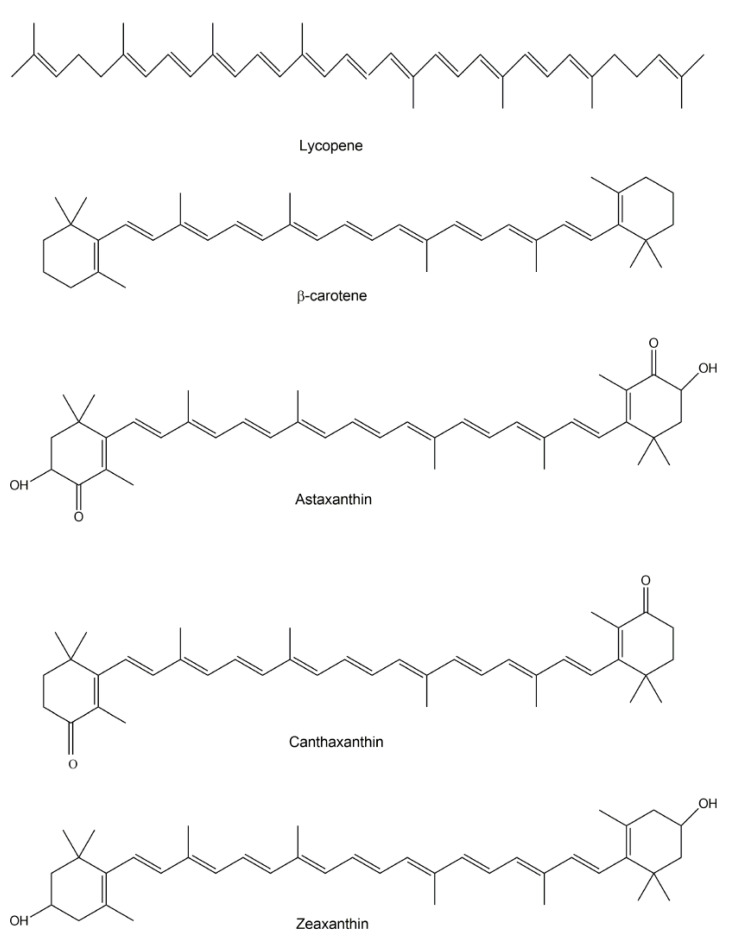
Structure of representative carotenoids.

Carotenoids contain an aliphatic polyene chain usually composed of eight isoprene units that include light-absorbing conjugated double bonds, giving rise to characteristic colors such as yellow, orange, or red [14,15]. Representative structures of carotenoids are shown in Figure 1. Some carotenoids are precursors of vitamin A, specifically those with β-ring end groups, such as β-carotene, zeaxanthin, and β-cryotoxanthin [8]. From the standpoint of human health, carotenoids are among the bioactive products with significant medical value. For example, carotenoids have been found to lower the risks of diseases, including cancer, cardiovascular diseases, and age-related eye disorders, such as macular degeneration and cataracts [16].

Carotenoid-producing fungi are very diverse, including *Rhodotorula mucilaginosa* [17], *Blakeslea trispora, Phycomyces blakesleeanus, Mucor circinelloides, Fusarium sporotrichioides* [18], *Rhodosporium paludigenum*, and *Rhodotorula glutinis* [19]. Carotenoids protect fungi from oxidative stress and non-ionizing irradiation such as UV light. In addition, carotenoids are intermediates essential for synthesizing other biological molecules including bioactive apocarotenoids and related constituents. For instance, in many filamentous fungi, retinol, the precursor of vitamin A as well as an essential component of rhodopsins (membrane-bound photoreceptors), is produced via the oxidative cleavage of β-carotene [20]. In Mucorales, β-carotene is an intermediate during the synthesis of trisporoids, apocarotenoid derivatives that include the sexual hormones the trisporic acids. In addition, trisporoids have been proposed as substrates in the synthesis of sporopollenin, one of the most chemically inert biological polymers primarily found in the tough outer walls of plant spores and pollen grains [15]. In some fungi, such as the filamentous species *Blakeslea trispora* [21] and the yeast *Rhodotorula* spp. [22], the β-carotene biosynthetic pathway also produces lycopene, a carotenoid intermediate. Lycopene is a nutrient with antioxidant properties. It was originally identified in plants such as tomato and watermelon, giving their fruits the characteristic red and pink colours. Lycopene has been linked to several human health benefits ranging from improved cardiovascular health to protection against sunburns and certain types of cancers.

Aside from β-carotene and lycopene, another fungal carotenoid, astaxanthin, has attracted significant attention for its promise in the pharmaceutical, food and cosmetics industries. For example, astaxanthin has shown promise for treating Alzheimer’s disease, Parkinson’s disease, stroke, high cholesterol, age-related macular degeneration (age-related vision loss), and preventing cancer. *Xanthophyllomyces dendrorhous* (synonym *Phaffia rhodozyma*), a red- or orange-pigmented basidiomycetous yeast initially isolated from Japanese and Alaskan tree-exudates decades ago, is capable of producing astaxanthin [23,24]. Astaxanthin is synthesized via the mevalonate pathway starting with a condensation of two molecules of geranylgeranyl pyrophosphate, a four-step desaturation, cyclization, and a final 4-ketolation plus 3-hydroxylation [25,26]. In addition to astaxanthin, *X. dendrorhous* can also utilize the mevalonate pathway to produce several other types of carotenoids including β-carotene, canthaxanthin, and zeaxanthin. Canthaxanthin is used to reduce sensitivity to sunlight (photosensitivity) experienced by people who have a rare genetic disease called erythropoietic protoporphyria (EPP) [27]. In these people, sunlight can cause skin reactions such as rash, itch, and eczema. Similarly, zeaxanthin also protects human cells from the harmful effects of certain light sources such as the Sun. However, unlike canthaxanthin, that is preferentially deposited in the skin, zeaxanthin is an eye pigment that, once inside the body, accumulates in the eyes and helps to build a yellow-colored pigment shield to protect the eye cells. Indeed, the ability of *X. dendrorhous* to produce such a diversity of bioactive carotenoids makes it a potential model host to be engineered as a cell factory for the production of medically and industrially important carotenoids [28].

### 2.2. Melanin

Broadly speaking, melanins are heterogeneous polymers derived by the oxidation of phenols and subsequent polymerization of intermediate phenols and their resulting quinones. Fungal melanins are a subset of natural melanins and are polyphenolic and/or polyindolic compounds with high molecular masses and negative charges. Fungal melanins can appear dark green, brown, or black. In fungal cells, melanin serves as an antioxidant and a scavenger of reactive nitrogen species (RNS) and reactive oxygen species (ROS). Commonly found in pathogenic fungi, melanin pigments are thought to provide those pathogens protection from UV and other radiation and aid in evasion from the host’s immune system attacks [29]. Fungal melanins belong to three major types: 1,8-dihydroxynaphthalene (DHN) melanin, 3,4-dihydroxyphenylalanine (DOPA)-melanin (also called eumelanin), and pyomelanin [30,31]. DHN melanin is produced from acetyl-coenzyme A via the polyketide synthase pathway. The fungus *A. fumigatus* is able to synthesize DHN melanin, which contributes to the gray-green appearance of its conidia. The deletion of its polyketide synthase PksP in *A. fumigatus* gave rise to colorless spores with weakened virulence [32]. Eumelanin is converted from *o*-diphenolic or *p*-diphenolic substrates by a polyphenol oxidase (laccase). In *Candida albicans*, eumelanin particles are observed in vitro and in infected mammalian tissues, such as murine kidney and human skin [33]. In this ascomycete fungus, melanin is externalized in the form of electron-dense melanosomes that are extracellularly secreted or loosely attached to the cell wall surface through binding with chitins [34]. Although *C. albicans* has laccase activity [33], the association of melanin with pathogenicity remains to be clarified for this fungus. Pyomelanin is an extracellular water-soluble pigment, in contrast to both DHN- and DOPA-melanins that are cell wall-immobilized and insoluble in water [31]. Pyomelanin is produced by the polymerization of homogentisic acid degraded from L-tyrosine/L-phenylalanine. In *A. fumigatus*, pyomelanin plays an important role in the germination of conidia and in the defensive responses toward external oxidative stresses [31].

Apart from the above-stated three major types of fungal melanins, there is another pathway of melanin biosynthesis in mushrooms such as the common cultivated mushroom *Agaricus bisporus* and others [35]. In these mushrooms, melanin is formed from a benzoquinone. Benzoquinone is converted from the precursor γ-glutaminyl-4-hydroxybenzene (GHB) by tyrosinase. GHB is the main phenolic compound in *A. bisporus* fruiting bodies and spores [36].

In addition to its involvement as a virulence factor in fungal pathogens, melanin enables fungi to survive and thrive in adverse and even extreme environments, providing protection against desiccation, non-ionizing (i.e., UV light) and ionizing radiation, as well as oxidative and nitrosative stresses [37,38]. Over the past decade, melanin pigments and their subunits have kindled great interest as soft biocompatible functional materials with antioxidant characteristics for engineering high-performance, low-impact biocompatible optoelectronic devices, such as light emitting diodes, memory devices, etc. [39]. Intriguingly, halophilic fungal isolates of two species, *Trimmatostroma salinum* and *Phaeotheca triangularis,* from the eastern coast of the Adriatic Sea, have been found to produce melanin in sodium chloride solutions at saturated concentrations [40]. Similarly, high-dose radiation resistant melanin was found in an Antarctic desert-dwelling (or desert-inhabiting) fungus, *Cryomyces antarcticus*. These fungal extremophiles could serve as promising sources of melanin with excellent physicochemical properties for a variety of industrial and medical applications.

### 2.3. Polyketides

Many fungal pigments are polyketide-based compounds. This group of fungal pigments is abundantly produced by most ascomycetous fungi, including the filamentous ascomycete genera *Neurospora* and *Monascus* [41].

Fungal polyketide pigments are made of tetraketides and octoketides which have eight C_2_ units giving rise to the polyketide chain. Anthraquinones (including hydroxyanthraquinones), and naphthoquinones are representative classes which display an arsenal of various colors [42,43]. The basic structure of the anthraquinone class of pigments is a polycyclic aromatic hydrocarbon which is derived from anthracene or phthalic anhydride, i.e., the merger of three benzene rings. Anthraquinone itself is highly insoluble and is generally used in the manufacture of dyes for the textile and pulp industries [44,45]. However, other members of the anthraquinone class have been used in the pharmaceuticals industry.

Approximately 700 representatives of anthraquinones have been found in fungi, plants, and lichens, conferring a yellow, orange, or brown color to the mycelium of microscopic fungi, the fruiting bodies of macroscopic fungi (mushroom), as well as lichens [46]. The variety of anthraquinones results from the presence of different substituents, like -OH, -CH_3_, -OCH_3_, -CH_2_OH, etc. as well as the reduction of carbonyl groups or double bonds in the benzene ring. Anthraquinones produce a yellow color, whereas the substituents produce various hues of the molecules ranging through yellow, orange, red, brown, and violet [47]. The most widely distributed variants found among fungi are 1, 8-dihydroxy and 1, 5, 8- or 1, 6, 8- trihydroxy anthraquinone derivatives [48]. However, substituents at other positions of the ring have been recently identified. For example, chrysophanol is a trihydroxyanthraquinone with a methyl substituent at C-3. It has shown antiviral and anti-inflammatory activities. Both chrysophanol and helminthosporin have shown inhibitory activity against cholinesterase with helminthosporin showing permeability across the blood-brain barrier [49]. Cholinesterase is a key enzyme involved in the development of Alzheimer’s disease. Most anthraquinone-producing fungi produce a mixture of pigments of the quinone class. *Aspergillus crisfafus*, for instance, has been found to synthesize as many as 15 pigments related to anthraquinone. A strain of *Curvularia lunata* has been characterized that produces a mixture of three anthraquinone derivatives: chrysophanol, helminthosporin, and cynodontin, with cynodontin (1, 4, 5, 8-tetrahydroxy-3-methylanthraquinone) comprising more than 70% of the mixture and showing potential as a pharmaceutical with antifungal activity [44].

Chrysophanol, helminthosporin, and cynodontin are all hydroxyanthraquinones (HAQNs), derivatives of anthraquinone via the substitution of hydrogen atoms by hydroxyl groups plus the addition of other functional groups. Anthraquinones including HAQNs have been found to be produced by such fungi as *Aspergillus* spp., *Eurotium* spp., *Fusarium* spp., *Dreschlera* spp., *Penicillium* spp., *Emericella purpurea*, *Culvularia lunata*, *Mycosphaerella rubella*, *Microsporum* spp., etc. [48].

Filamentous fungi, such as *Penicillium* and *Aspergillus*, synthesize HAQNs through the polyketide pathway. This process has been employed for the production of natural food-grade colorants [50]. Apart from their usage as colorants in the food and cosmetics industries, they have been found to possess antiviral activities. For instance, emodin has been extracted from *Penicillium citrinum, P. islandicum*, *Aspergillus glaucus* and *A. variecolor* [51,52] and aloe-emodin from *Penicillium oxalicum* [53]. Emodin and its congener aloe-emodin are known antiviral agents [54]. Aspergilol H and I, HAQNs produced by *Aspergillus versicolor*, have been reported to have antiviral effects against HSV-1 [55]. Notably, HAQNs are able to absorb visible light and display colour, with the colours varying based on the position and number of the hydroxyl substituents in the different rings [56]. HAQNs are relatively stable and they exhibit superior brightness in comparison to azo pigments.

Naphthoquinone is a class of organic compounds structurally related to naphthalene. Naphthoquinones have very significant pharmacological activities against bacterial, fungal, viral, and insect pathogens and pests. In addition, they have anti-inflammatory, and antipyretic properties. *Fusarium* spp. have shown capable of producing a diversity of naphthoquinones with a broad spectrum of biological activities [57,58]. For instance, a compound produced by *Fusarium fujikuroi* and other fungi, bikaverin, a red heterotetracyclic pigment with the chemical structure of 6,11-dihydroxy-3,8-dimethoxy-1-methylbenzo[b]xanthene-7,10,12-trione, has shown anti-neoplastic activity, suggesting its potential as a pharmaceutical agent against lymphoma, carcinoma, and sarcoma, etc. [59,60,61,62].

### 2.4. Azaphilones

**Figure 2:**
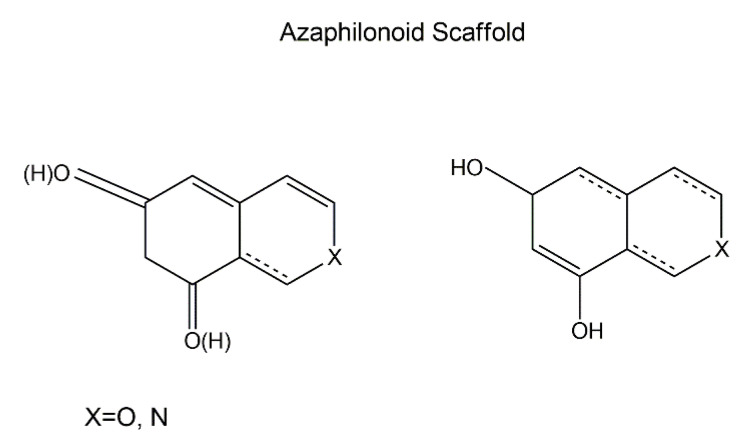
Azaphilonoid scaffold.

**Figure 3:**
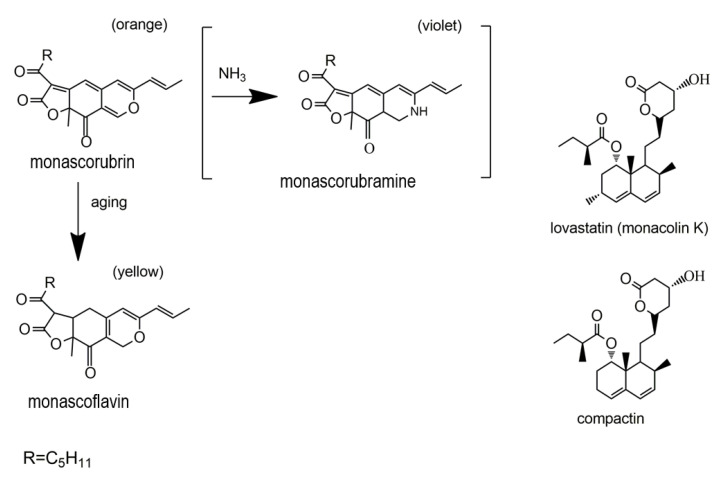
Azaphilone pigments derived from the yeast *Monascus*.

Azaphilones are a structurally variable group of fungal secondary metabolites (polyketide derivatives) [63], namely pigments with a highly oxygenated pyrone-quinone bicyclic core usually known as isochromene and a chiral quaternary centre [64,65]. Both a pyrone-quinone backbone and a chiral quaternary centre (Figure 2) are essential for compounds to be classified as azaphilones [66]. Azaphilone is so designated because of its ready insertion of nitrogen. For example, ammonia readily converts the pyran oxygen of monascorubrin into the violet-colored nitrogen analog monascorubramine (Figure 3) [67].

The azaphilone pigments are produced by a plethora of the ascomycetous and basidiomycetous fungi, particularly the former [68], including the genera *Aspergillus*, *Penicillium*, *Chaetomium*, *Talaromyces*, *Pestalotiopsis*, *Phomopsis*, *Emericella*, and *Epicoccum,* as well as *Monascus* and *Hypoxylon*, where they give rise to the yellow, red, or green hues of fruiting bodies and/or mycelia [69].

Fungal azaphilones, structurally bearing the isochromane-like ring, possess a broad-spectrum of biological activities including monoamine oxidase inhibition, phospholipase A2 inhibition, tumor promotion suppression, gpl20-CD4 binding inhibition, and acyl-CoA: cholesterolacyltransferase (AGAT) inhibition. However, the relationships between the structures of azaphilones, their spectrum of activities, and the corresponding mechanistic modes of action remain elusive [66].

**Figure 4:**
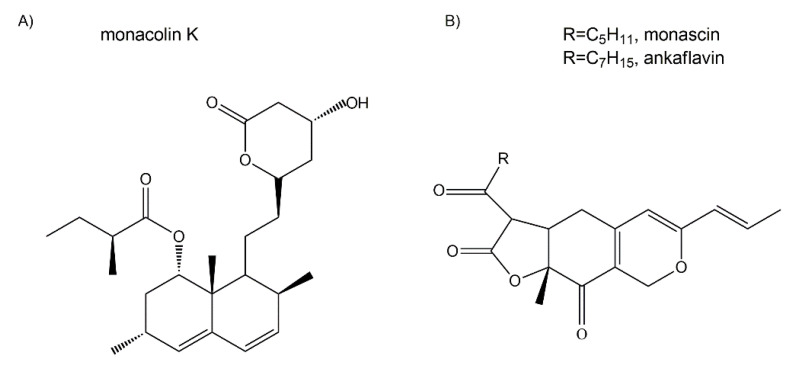
Chemical structure of monacolin K, monascin and ankaflavin. (**A**) Monacolin K; (**B**) Monascin and ankaflavin.

Monascin and ankaflavin (Figure 4B) are two yellow pigments of the azaphilonoid group. The fungal genus *Monascus*-derived monascin and ankaflavin have been found to potently inhibit preadipocyte differentiation, and promote lipolysis of mature adipocytes [70]. Furthermore, these two pigments are able to reduce blood lipids as well as lower the synthesis and accumulation of triglycerides (TG) [71]. For example, Lee et al. (2013) investigated the effects of eight-week administrations of monascin and ankaflavin on obesity factors using obese rats fed with a high-fat diet [72]. Their work illustrated the involvement of these two *Monascus* azaphilones in the modulation of preadipocyte differentiation, lipogenesis, and lipid absorption, highlighting their potential in the development of hypolipidemic drugs.

Monacolin K (viz. lovastatin) is a statin compound and previously thought to be a hypolipidemic component of *Monascus*-fermented products. While earlier studies suggested anti-obesity effects of monacolin K (Figure 4A), recent investigations indicated that monascin and ankaflavin (Figure 4B), rather than monacolin K, were likely the major players in the *Monascus*-mediated amelioration of diabetes and fatty liver [73].

Indeed, there is mounting evidence that a long-term high-dosage administration of monacolin K might lead to numerous side effects such as rhabdomyolysis and reduction in the level of coenzyme Q_10_ (CoQ_10_). Furthermore, although monacolin K reduces the serum total cholesterol (TC), triglycerides (TG) and low-density lipoprotein cholesterol (LDL-C) levels, it also substantially reduces high-density lipoprotein cholesterol (HDL-C) levels. On the other hand, Lee et al. (2010) showed that monascin and ankaflavin could increase HDL-C levels significantly while imposing no damage on the liver or kidneys [68]. In addition, monascin has been shown to protect the liver from chemical damage via its anti-inflammatory effect [74]. Other work with monascin has indicated that it significantly inhibits peroxynitrite (ONOO^−^; PN), and ultraviolet light B (UVB)-induced skin carcinogenesis [75].

Together, the above results suggest that monascin and ankaflavin possess hypolipidemic, anti-atherosclerosis, antioxidative, and anti-inflammatory activities, thereby rendering them promising compounds that may be of value in the prevention and treatment of cardiovascular diseases [73].

Apart from *Monascus* species, the production of azaphilone pigments by other fungal species has also been documented [76]. For instance, chaetoviridins, a class of azaphilones synthesized by *Chaetomium globosum*, have been found to have strong antifungal activities. To date, four chaetoviridins, chaetoviridin A―D, have been characterized. Studies with mice have demonstrated that chaetoviridin A is able to suppress tumor progression via 12-O-tetradecanoylphorbal-13-acetate in two-phase carcinogenesis [77]. Independent work by Tomoda et al. revealed that chaetoviridin A and B might exert inhibitory effects on cholesteryl ester transfer protein [78].

### 2.5. Other Fungal Pigments

**Figure 5:**
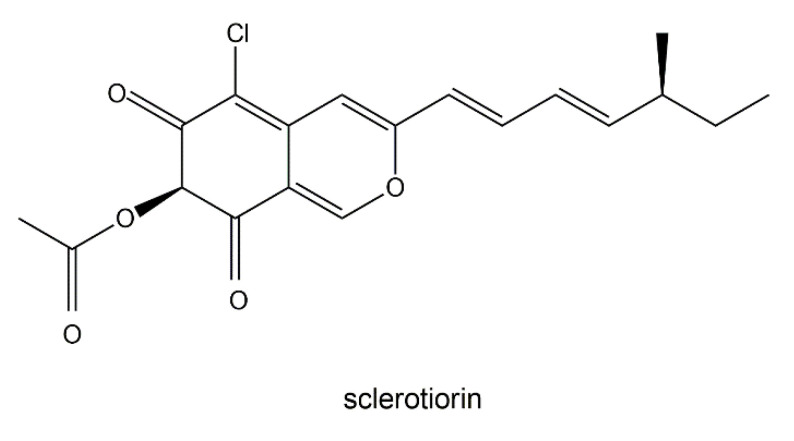
Chemical structure of the pigment sclerotiorin produced by *Penicillum sclerotiorum* 2AV2.

The pigment sclerotiorin has been isolated from cultures of *Penicillum sclerotiorum* 2AV2, which was recovered from Amazonian soil [6]. Sclerotiorin (Figure 5) has been found to have a variety of biological activities. Some of the biological activities most pertinent to human health include inhibitory effects on aldose reductase [79], endothelin receptor binding activity [80], antimicrobial effects including antifungal activity [17,81], apoptosis-triggering actions towards HCT-116 cancer cells [82], and the inhibition of integrase and protease of HIV-1 [83].

Studies on *Penicillum chrysogenum* have identified a polyketide synthase (PKS) gene required for the biosynthesis of sorbicillinoid, a yellow pigment [84]. The authors demonstrated that a highly reducing PKS enzyme, encoded by Pc21g05080 (*pks13*), is indispensable for the production of polyketide precursors such as sorbicillinol and dihydrosorbicillinol as well as their derivatives such as bisorbicillinoids. PKSs are also involved in the biosynthesis of sorbicillinoid in *Acremonium chrysogenum*, the fungal species used in the industrial production of cephalosporin C, an antibiotic in current clinical use [85].

## 3. Biosynthesis of Fungal Pigments

So far, four major pathways have been identified as responsible for the biosynthesis of the main fungal pigments, including the polyketide synthetic pathway, the shikimate pathway, the terpenoid synthetic pathway, and the nitrogen-containing metabolite pathway.

### 3.1. Polyketide Synthetic Pathways

**Figure 6:**
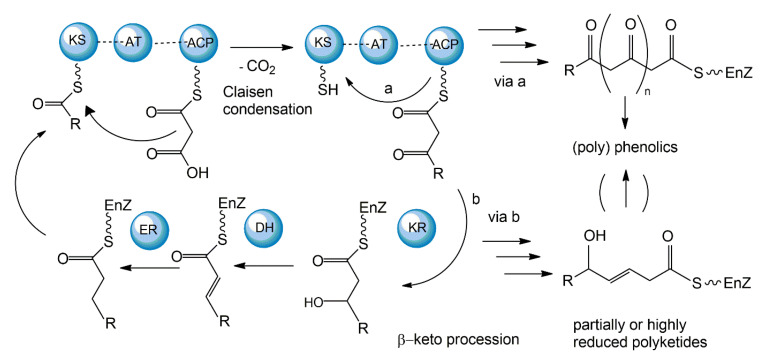
Mechanisms of fungal polyketide biosynthesis.

The polyketide synthetic pathway is involved in producing many fungal pigments with human health relevance. For example, there is increasing evidence that fungal pigments such as melanin, quinones, flavins, ankaflavin, and azaphilones, all relevant to human health [5,6], involve the polyketide synthesis pathway [86,87]. This pathway has been studied in diverse fungal species, including those in the genera *Monascus, Fusarium*, *Alternaria*, and *Epicoccum* [64]. The core polyketide synthetic pathway is shown in Figure 6. Fungal pigments are produced by this pathway via repetitive Claisen condensations of an acyl-coenzyme A (CoA) starting unit with malonyl-CoA elongation units in a manner analogous to fatty acid biosynthesis. The polyketide pathway generates either aromatic ketides or fatty acids. The extending chain of the aromatic ketides is stabilized by cyclization reactions and partial reduction. However, in the fatty acids, the carbonyl groups of the chain are reduced prior to the addition of the next C_2_ group. One of the major differences between the two metabolic routes is that polyketides have varied degrees of β-keto processing. Specifically, some are not or only partially reduced, giving rise to the formation of (poly-)cyclic aromatic compounds. On the other hand, they may be significantly reduced, yielding linear or macrocyclic, non-aromatic carbon skeletons.

Aromatic ketides of this pathway include tetra-, hepta-, octa- and higher number ketides. Fungi possess a variety of pigments of the octaketide origin based on the anthra-9,10-quinone backbone with both rings being substituted. Anthraquinones are representatives of this type of pigment. In many cases anthraquinones are found in fungi in the corresponding colourless reductive states (anthranol, anthrone, anthrahydroquinone, and oxanthrone derivatives) that may be present in the forms of diverse glycoside conjugants. However, many naturally occurring, coloured anthraquinones are oligomers made by the coupling of two or more anthraquinone molecules. These oligomers differ in the number and positions from which monomers and amino acids are ligated to generate a diversity of fungal pigments [88,89].

A large number of enzymes have been identified in the fungal polyketide synthesis pathway. These include a core set of enzymes, including ketosynthase (KS), acyl transferase (AT), and acyl carrier protein (ACP) domains. Further, optional β-keto processing steps may be catalyzed by keto reductase (KR), dehydratase (DH) and enoyl reductase (ER) domains. Other optional ancillary domains involve cyclase (CYC) [90] and methyl transferase (MT) activities [91]. Based on their architecture and the presence or absence of additional β-keto processing domains, fungal PKSs are categorized into non-reducing or aromatic (NR-PKS), partially reducing (PR-PKS), and highly reducing PKS (HR-PKS).

The synthesized fungal polyketides can vary in their chain length, the degree of β-keto processing, and cyclization. Moreover, the tremendous structural diversity of polyketides is further obtained from derivatization of the polyketide carbon backbone by alkylation, acylation, and oxygenation, and by post-PKS modification or tailoring. Genome sequence analyses so far have revealed that all genes essential for fungal polyketide biosynthesis are clustered, including the PKS genes, genes encoding enzymes associated with tailoring, as well as regulatory genes [92].

While most fungal polyketide pigments are synthesized as aromatic ketides, the biosynthesis of azaphilones uses both the polyketide pathway and the fatty acid synthesis pathway. The polyketide pathway assembles the main polyketide chain of the azaphilone pigments from acetic acid (the starter unit) and five malonic acid molecules (the chain extender units) in a conventional way to generate the chromophore structure. The fatty acid synthesis pathway produces a medium-chain fatty acid (octanoic or hexanoic acid) that is then bound to the chromophore by a transesterification reaction [93,94].

### 3.2. Shikimate Pathways

Aromatic amino acids act as the precursor of various fungal secondary metabolites such as pigments and vitamins [95]. In the fungal phylum *Basidiomycota*, tyrosine is the precursor of a distinct class of pigments, the betalains, solely present in the genera of *Amanita* and *Hygrocybe* [96,97]. In the phylum *Ascomycota*, tyrosine-derived pigments, such as melanin and tyrosine betaine, are thought to contribute to stress tolerance (e.g., temperature, radiation) [98,99] and pathogenicity [100,101].

The shikimate pathway is present in the prokaryotes, microbial eukaryotes and plants studied. However, it is absent in those animal species investigated [102]. This pathway links carbohydrate metabolism to the biosynthesis of aromatic compounds. Metabolically, the shikimate pathway is a seven-step route utilized by fungi for the biosynthesis of aromatic amino acids like phenylalanine (Phe), tyrosine (Tyr), and tryptophan (Trp), as well as para-aminobenzoic acid, via the central intermediates shikimic and chorismic acids [86]. The first step involves the condensation of the glycolytic intermediate phosphoenol pyruvate and pentose phosphate pathway intermediate erythrose-4-phosphate to yield a seven-carbon heterocyclic compound, 3-deoxy-D-arabinose-heptulosonate-7-phosphate derivative (DAHP). The second step involves the generation of a highly substituted cyclohexane derivative, 3-dehydroquinate, by the replacement of the exocyclic C_7_ of DAHP by the ring oxygen. The remaining five steps involve the introduction of a side chain and two of the three double bonds that convert this cyclohexane into a benzene ring, the core of aromatic amino acids [5]. The metabolic routes may vary for different classes of pigments.

The pigments produced via the shikimate pathway are generally water-soluble phenolic compounds, including terphenyls and pulvinic acids. Studies of *p*-terphenyls as a family of the mushroom pigments were initiated in 1877. Isolation of polyporic acid, atromentin and thelephoric acid represented the inception of the chemical investigation of fungal pigments. The elucidation of the structures of polyporic acid and atromentin by Kögl is recognized as a milestone in organic chemistry [103]. It has been demonstrated that some terphenyls display biological activities, such as immunosuppressive, antithrombotic, anticoagulant, neuroprotective, 5-lipoxygenase inhibitory (for the treatment of inflammatory bowel disease), and cytotoxic activities [104].

**Figure 7:**
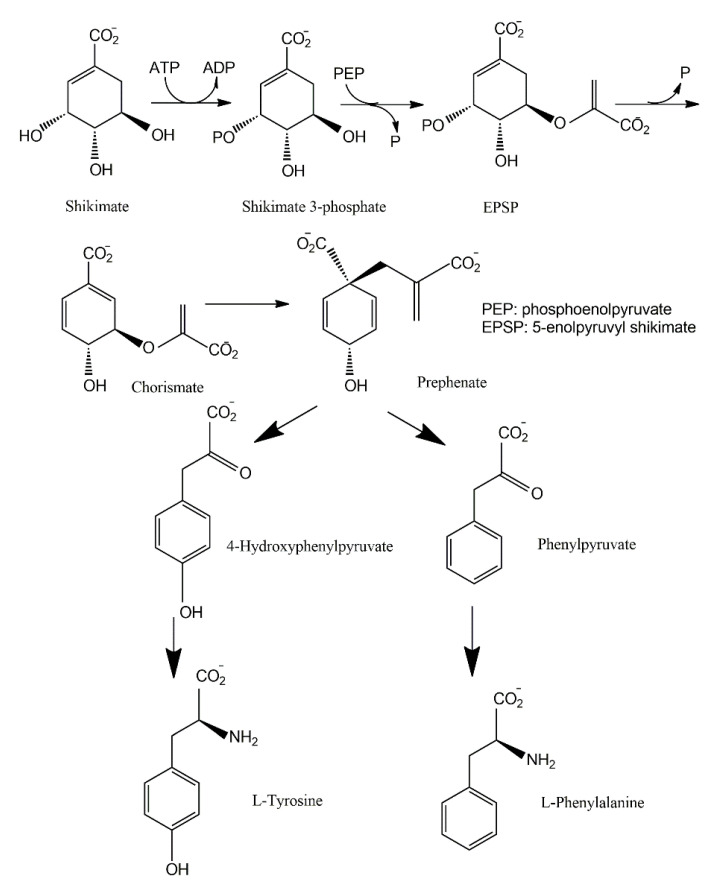
Shikimate pathway leading to biosynthesis of *p*-terphenyls.

Many fungi have evolved pathways utilizing the aromatic products of shikimate metabolism (Figure 7). The initial steps in the biogenesis of *p*-terphenyls are the well-known reactions of primary metabolism flowing from shikimate to chorismic acids and subsequently to arylpyruvic acids. Experiments involving the feeding of ^13^C- and ^14^C-labeled precursors to fungal cultures revealed that *p*-terphenyls are assembled by initial condensation between two molecules of unbranched phenylpropanoid precursors, either phenylpyruvic acid or phenylalanine. Previous studies also revealed the involvement of 4-hydroxyphenylpyruvic acids or tyrosine in the initial condensation during the biosynthesis of terphenylquinones such as atromentin [105]. Atromentin is known as a key intermediate for further conversions, for instance, to more highly hydroxylated terphenylquinones and pulvinic acids [106,107].

### 3.3. Terpenoid Synthetic Pathways

**Figure 8:**
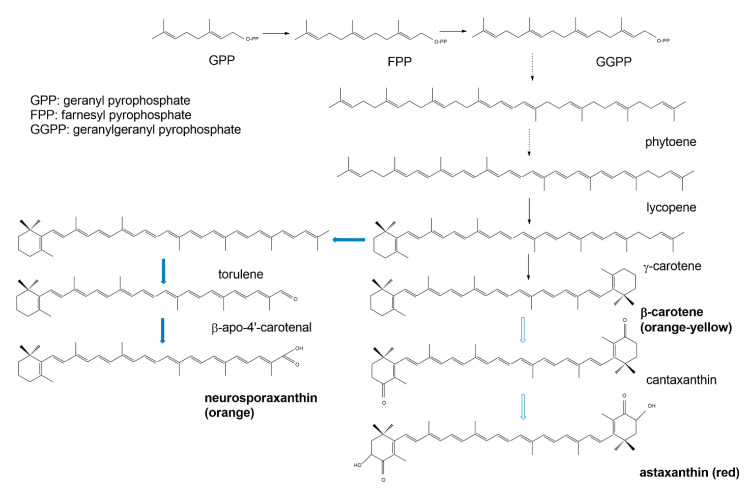
Simplified scheme of the biosynthetic pathways for β-carotene (black arrows) and the astaxanthin and neurosporaxanthin (blue arrows) from GPP.

Biochemically, the carotenoids are terpenoids [15]. Accordingly, the synthesis of carotenoids utilizes the terpenoid synthetic pathways deriving from the condensation of C_5_ isoprene units [108]. The common terpenoid precursor is isopentenyl pyrophosphate (IPP), which is synthesized either via the mevalonate pathway (generated from hydroxymethylglutaryl coenzyme A, HMG-CoA), or via the non-mevalonate pathway (generated from the condensation of pyruvate and glyceraldehyde 3-phosphate [109]. So far, available research has demonstrated that fungal IPP is produced via the mevalonate pathway, whereas in bacteria and photosynthetic species IPP is produced via the non-mevalonate pathway. The early biosynthetic steps involve the sequential additions of IPP (isoprene, C_5_) units to yield geranyl pyrophosphate (GPP, C_10_), farnesyl pyrophosphate (FPP, C_15_), and geranylgeranyl pyrophosphate (GGPP, C_20_) [108,109]. The initial compound possessing the typical aliphatic carotenoid-like structure is 15-cis-phytoene (a colorless molecule), consisting of a symmetrical polyene chain generated via the condensation of two GGPP units catalyzed by phytoene synthase (Figure 8). The light-absorbing characteristics of carotenoids are attributed to the presence of a chromophore, which is comprised of an array of conjugated double bonds via the catalysis of desaturases. Different subsequent chemical modifications, usually the introduction of a cyclic end group (with β and ε rings being the most frequently introduced) into at least one of the ends of the molecule, and/or oxidative reactions (for instance, hydroxylation, carboxylation, epoxidation, esterification, etc.), may contribute to the huge arsenal of carotenoids [108].

### 3.4. Nitrogen-Containing Metabolite Pathways

**Figure 9:**
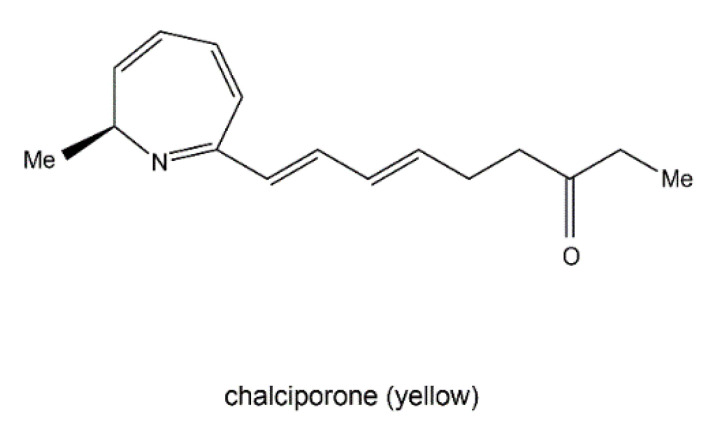
Chalciporone.

Chalciporone (Figure 9) is a type of 2H-azepine alkaloid pigment produced by the mushroom *Chalciporus piperatus* (Basidiomycetes). This pigment is believed to act as a deterrent to insects and other predators and thereby can potentially protect the mushroom. The biosynthesis of chalciporone was illustrated in 2001 [110]. In their experimentations, the radioactive labelled sodium [U-^13^C_2_] acetate was not incorporated into chalciporone by *C. piperatus*. In contrast, the supplementation of a mixture of [U-^13^C]-labelled fats led to chalciporone, in which seven intact acetate units were incorporated (*ca.* 10 atom % enrichment) between C_3_ and C_16_. However, the enrichment of the C_1_ methyl group and the adjacent carbon (C_2_) was not observed during this experiment, suggesting that the CH_3_–CH–N moiety was derived from an α-amino acid. This was confirmed by the observation that administration of a mixture of [U-^13^C]-labelled amino acids to *C. piperatus* gave rise to the enrichment of both C_1_ and C_2_ and of all the other carbon signals in the spectrum of chalciporone [107]. The work clearly demonstrated that the carbon backbone of chalciporone is generated from an amino acid plus seven acetate (=malonate) units.

**Figure 10:**
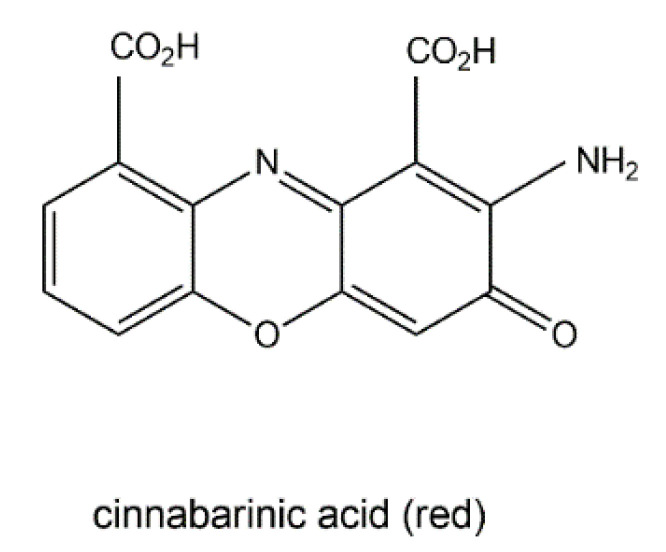
Cinnabarinic acid.

The wood-rotting fungus *Pycnoporus cinnabarinus* (Basidiomycetes) can produce a red pigment, cinnabarinic acid (Figure 10), via oxidative dimerization of the precursor 3-hydroxyanthranilic acid in sporocarps as well as in culture broth [111]. This reaction is catalyzed by laccase and necessary for the production of antibacterial compounds by the fungus. Cinnabarinic acid shows inhibitory effects towards several Gram-positive bacteria of the *Streptococcus* genus. It is known that cinnabarinic acid shares structural homology with the antibiotic group of actinomycins (e.g., actinomycin D) produced by *Streptomyces* spp., having two cyclic pentapeptides linked to the phenoxazinone chromophore.

**Figure 11:**
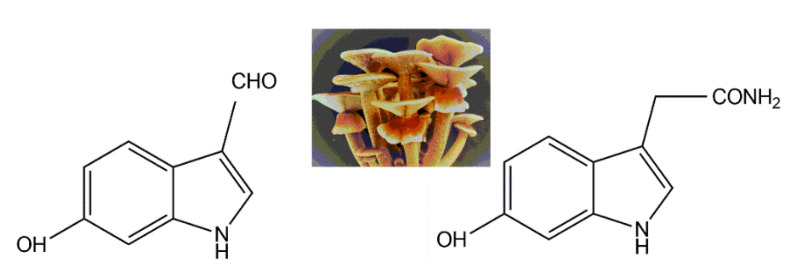
The indolic compounds 6-hydroxy-1H-indole-3-carboxaldehyde (**left**) and 6-hydroxy-1H-indole-3-acetamide (**right**) isolated from the fruiting bodies of *Agrocybe cylindracea* (middle, insert image).

Two indole pigments were isolated as free radical scavengers from the fruiting bodies of the mushroom *Agrocybe cylindracea* [112]. Based on spectroscopic data, they were identified as 6-hydroxy-1*H*-indole-3-carboxaldehyde and 6-hydroxy-1*H*-indole-3-acetamide (Figure 11). 6-hydroxy-1*H*-indole-3-acetamide is an amide derivative of 6-hydroxyindole-3-acetic acid. A previous study identified that 6-hydroxyindole-3-acetic acid could be produced through the transformation of indole-3-acetic acid by *Aspergillus niger* [113]. These two indolic compounds were found to possess potent inhibitory activity on lipid peroxidation in rat liver microsomes, with IC_50_ values of 4.1 and 3.9 μg per ml, respectively [109].

## 4. Interaction of Fungal Pigments with the Host Immune System

One of the most interesting defense mechanisms of fish may involve non-specific immune responses that have evolved due to their long-term associations with fungi [114]. Specifically, the prevalent colonization of *Rhodotorula mucilaginosa* (a red yeast) in the fish gastrointestinal (GI) tract might have contributed to the stimulation of non-specific mechanisms of host immunoprotection as well as the maturation of the host GI tract [115]. Spectrophotometric analysis in wild fish (*Abramis brama*, *Rutilus rutilus*, *Perca fluviatilis*) indicated the presence of a set of three pigments—β-carotene, torularhodin, and torulene, which are produced by the commensal *Rh. mucilaginosa*. In addition to acting as scavengers of free radicals, these fungus-derived pigments are hypothesized to play a role in the immunostimulation of the host via enhanced activation of T lymphocytes, supporting the action of macrophages [116] or antimicrobial activity [115].

For decades, an accumulating body of evidence has revealed that carotenoids or their derivatives are involved in the activation of thymocites [117], the expression of immune-associated genes [118], and the increase in membrane fluidity [119], which represent vitally important functions in mounting immune responses in animals. Notably, the immunostimulatory effects of carotenoids are thought to be distinct from their antioxidant property [116]. Earlier investigations indicated that β-carotene and the non-provitamin A carotenoids are able to enhance cell-mediated and humoral immune responses in mammals [116]. It is not surprising that β-carotene-producing red yeast *Rh. mucilaginosa* might be beneficial to its hosts, in this case the wild fish, presumably through immuno-stimulation.

Given that (i) immunostimulatory effects of carotenoids in animals are thought to be independent of their antioxidant property [116], and (ii) innate immunity relies on effectors which produce cytotoxic molecules that may not only kill pathogens, but also harm host tissues, the mechanisms underlying the roles of carotenoid pigments in the well-being of animals need to be elucidated. Although the functionality of dietary antioxidants in invertebrate immunity is not fully understood, it has been found in vertebrates that carotenoids can scavenge cytotoxic radicals formed during the immune response. Carotenoids may consequently decrease the self-harming cost of immunity. A positive correlation between the effectiveness of the innate immune defense and the levels of circulating carotenoid might therefore be expected. In accordance with this hypothesis, a study using the amphipod species *Gammarus pulex* showed that the maintenance and use of the prophenoloxidase system were highly correlated with the carotenoid level in the haemolymph within the natural populations of this crustacean [120].

The enhanced immunity by carotenoids has also been demonstrated in mammals [121] where mice administered *Rhodotorula glutinis-*derived carotenoid could survive for 2 weeks post lethal-dose challenges of pathogenic *Pseudomonas aeruginosa* and *C. albicans.* This result points to the potential use of fungal carotenoids as food additives or dietary supplements to boost the human immune response against microbial infections.

Like carotenoids, melanins are ubiquitously produced by fungi, particularly pathogenic fungi such as *Cryptococcus neoformans*, *Aspergillus fumigatus*, and *Candida albicans*. As described previously, melanins in fungal pathogens contribute to fungal pathogenesis towards mammalian cells [10]. The melanin pigments contribute to antigen masking to circumvent recognition by host immune system and to the survival of fungal pathogens against phagocytosis [122]. For example, phagocytosed *C. neoformans* produces melanin to protect against the oxidative environment inside the phagolysosome [122]. For *A. fumigatus*, it is believed that melanins protect the conidia against reactive oxygen species, mask the recognition of various *A. fumigatus* pathogen-associated molecular patterns (PAMPs) by the host, inhibit macrophage apoptosis and phagolysosome fusion, and attenuate the host immune response. The protective effect of melanin might also be due to its role in modifying the surface properties of conidia and vegetative cells by altering the charge and hydrophobicity, thereby reducing the effectiveness of the immunological functions of the host [123]. For example, in contrast to the wild type conidia, melanin-deficient mutant conidia can activate human DCs (dendritic cells) and subsequent cytokine production, leading to their elimination by the host immune system.

The protective effects of melanin for fungal pathogens have also been demonstrated for autophagy. Autophagy plays an important role in host immunity to microbial pathogens. The autophagy system targets pathogens in phagosomes, promotes phagosome maturation and prevents pathogen escape into the cytosol. Autophagy protein LC3 is believed to be a key player during phagocytosis [124]. During *Aspergillus* germination, exposure of PAMPs enables the activation of host LC3-associated phagocytosis (LAP), which promotes the killing and eradication of fungi. Shielding PAMPs from detection by pattern recognition receptors is a predominant strategy adopted by fungi for evasion from host immunity. Recent studies with *A. fumigatus* have demonstrated that LAP activation also requires the removal of fungal cell wall melanin, based on the genetic, biochemical or biological (germination) data [125]. Fungal melanin is known to inhibit the NADPH oxidase-dependent activation of LAP via excluding the p22phox subunit from the phagosome. Therefore, LAP blockade is a general trait of melanin pigments [126]. Physiologically, melanin-triggered LAP blockade is able to enhance fungal virulence.

## 5. Medical Relevance of Fungal Pigments

In extreme environments, fungi having pigmented cell walls in their spores and mycelia are able to tolerate dehydration–hydration cycles, thermal fluctuations, toxic metal-contamination, and high ultraviolet light (UV) radiation better than the moniliaceous fungi, which are unpigmented [127]. A remarkable example is the black fungus *Cryomyces antarcticus*, an extremophile initially recovered from Antarctic deserts and later found to be able to survive in the outer part of the International Space Station [38]. The wide geographical distribution of pigmented fungal species and their extraordinary ability to survive in hostile environments suggest that fungal pigments may be of value in a variety of fields, including radioprotection and biomedical applications.

In addition to their broad distribution and their ability to synthesize secondary metabolites such as medically significant pigments, many fungi are readily grown in lab conditions. The prospect of large-scale production attracts interest from both the pharmaceutical industry and fundamental science. The scope of focus includes species from a wide range of environments of marine origin, soil, as well as endophytic fungi from terrestrial and marine flora and endolichenic origin [127,128].

### 5.1. Medical Roles of Melanins

Recent studies with fungi capable of producing melanin (also called melanotic fungi) have revealed a wide spectrum of functions for this kind of pigment, ranging from drought and radiation resistance to increased virulence in fungal pathogens. For example, melanotic fungi are present in diverse environments including but not limited to radiation-contaminated soils (e.g., the Chernobyl nuclear power station and the surrounding soils), Antarctic deserts, ancient cave paintings, and spacecraft, etc. In fact, melanized fungi, including spores of *Aspergillus niger*, *Cladosporium herbarum*, *Ulocladium chartatum*, *Basipetospora halophile*, etc., have been reported to be able to grow on the Mir Space Station and International Space Station. Some edible mushrooms (Basidiomycetes) are rich in melanins as well. Melanins contain persistent free radical centers, as detected by electron paramagnetic resonance (EPR) spectroscopy. These free radical centers allow melanins acting as antioxidants to scavenge free radicals and to protect the fungi from oxidative stress and damage by ionizing radiation in both atmospheric and terrestrial environments [129]. In a recent study [130], Pacelli et al. compared the effects of densely-ionizing deuterons and sparsely ionizing X-rays on two melanized fungal species, namely the fast-growing pathogenic basiodiomycete *Cryptococcus neoforman*s and the slow-growing Antarctic rock-inhabiting *Cryomyces antarcticus* to their non-melanized counterparts. *C. antarcticus* showed more resistance to deuterons than *C. neoformans*, and similar resistance to X-rays was observed for both species. Melanin offered protection against high-dose (1.5 kGy) deuterons for both *C. neoformans* and *C. antarcticus* (*p* < 10^−4^). In addition, melanin protected *C. antarcticus* (*p* < 10^−4^) and probably *C. neoformans* against X-rays (0.3 kGy). The use of both XTT (2,3-bis(2-methoxy-4-nitro-5-sulfophenyl)25-[(phenyl-amino)carbonyl]-2H-tetrazolium hydroxide) and MTT (2-(4,5-dimethyl-2-thiazolyl)23,5-diphenyl-2H-tetrazolium bromide) assays in parallel helped define the location of the melanin-mediated electron transfer in the cells. Deuterons increased XTT activity in melanized strains of both species, while the activity in non-melanized cells remained unchanged or decreased. The opposite was observed with levels of ATP (the indicator of the metabolic activity of cells): upon exposure to deuterons, ATP decreased in melanized strains, but not in non-melanized ones. Larger and more distinct differences in both XTT and ATP were found in *C. neoformans* than in *C. antarcticus*. Further research by Pacelli et al. revealed that *C. antarcticus* could produce both 1,8-dihydroxynaphthalene (DHN) and L-3,4- dihydroxyphenylalanine (L-DOPA) melanins, which likely contribute to its ability to thrive under diverse stress conditions [131]. Together, these results indicate for the first time that melanin could protect both fast- and slow-growing fungi from high-dose radiation (deuterons in this case) under physiological conditions [130]. Indeed, the remarkable ability of melanin to confer resistance to radiation led to further research that revealed that melanized fungi could harvest radiation energy for their growth and reproduction. Broadly speaking, such an ability has both fundamental ecological significance and practical implications. Ecologically, the ability of melanotic fungi to make use of electromagnetic radiation for their physiological processes helps us understand energy flows in the biosphere. Practically, the understanding paves the way for potentially developing melanin-based radiation resistant materials and equipment. In astrobiology and space travel, melanin represents a new type of material with which to experiment for space travel [132].

The unique stress-resistance properties of *C. antarcticus*, a heavily melanized black fungus in the class Dothideomycetes of the Ascomycota phyla, were further demonstrated by its ability to survive not only the Antarctic desert but also the harsh outer space environment and cosmic radiation exposure [38,130]. Indeed, the mechanisms for how fungal melanin molecules confer resistance to extreme heat/cold stresses and high-dosage radiations have gained wide attention from scientists [133,134]. One of the protection mechanisms is the chelating properties of melanins towards biologically damaging free radicals generated by the physical and chemical stresses. These results are inspiring biomedical engineering in healthcare and in radiation- and thermal-protective applications [135].

Aside from melanized microscopic fungi such as yeasts and molds, melanized macroscopic mushrooms are also attracting increasing attentions. One such mushroom is the medicinal mushroom *Inonotus obliquus* (also called Chaga), a basidiomycete. This mushroom grows mainly on birch trees and is broadly distributed across the northern hemisphere in Europe, Asia, and North America. Long used in folk medicine, Chaga contains massive amounts of melanin pigments. Several research groups have reported the therapeutic potential of Chaga’s bioactive pigments in countering the proliferation of cancer cells, and diabetes mellitus [136,137]. The pigments extracted from *I. obliquus* contain both phenolic compounds such as melanins, and triterpenoids such as inotodiol. Interestingly, the water-soluble component of melanin complexes of *I. obliquus* possesses insulin-sensitizing and hypoglycemic activity [138], consistent with their anti-diabetic functions. In addition, aqueous extracts of *I. obliquus* lowered the viability of human hepatoma (HepG2) cell lines in a dose-dependent fashion in vitro [139]. Subsequent studies revealed that the intraperitoneal administration of aqueous extracts of *I. obliquus* could inhibit tumor growth in vivo in mice implanted with melanoma B16-F10 cells at a dose of 20 mg/kg/d for 10 d [140]. Similarly, another study of melanin from *I. obliquus* showed its suppressive effect on the proliferation of HeLa 229 tumor cells [141]. Together with the above beneficial effects, the discovery of water-soluble melanin from *I. obliquus* in protecting DNA from carcinogenic damage [142] is also accelerating the preclinical studies of *I. obliquus* pigments. Results from such studies could generate significant medical and economic benefits in an environmentally friendly way (green chemistry).

As shown above, melanins have many potential benefits to humans. However, their presence in pathogenic fungi can also cause detrimental effects to humans both directly as human fungal pathogens or indirectly as plant and animal fungal pathogens. For example, in the human fungal pathogens *C. neoformans* [143], *Aspergillus fumigatus* [144], *Paracoccidioides brasiliensis* [145], *Penicillium marneffei* [146], *Fonsecaea pedrosoi* [147], and *Sporothrix schenckii* [148], melanin is an important virulence factor and contributes to drug resistance. Recently identified as a part of host innate immunity, platelets could be activated by exposure to *Aspergillus fumigatus*. In *A. fumigatus*, a mutant lacking melanin exhibited decreased platelet-stimulating activity, and the platelet activating effect can be mimicked by “melanin ghosts” [149], large isolated melanin molecules. Interestingly, melanins in *Fonsecaea monophora,* a common causal agent of chromoblastomycosis, were able to reduce Th1 cytokines and to elevate Th2 cytokines secreted by macrophages [150], likely by regulating the MAPK signalling pathway of macrophages [151], pointing out that melanins interacting with the macrophages might contribute to the immune evasion of fungal pathogen. In these species, melanin protects the invading fungal pathogen against oxidative stress within phagocytes, interferes with host cell signaling and autophagy, and masks recognition of fungal cells by the host immune system. Our understanding should benefit the development of therapeutic targets for treating mycosis in humans.

### 5.2. Medical Roles of Other Pigments

#### 5.2.1. Anti-Tumor Activities

Carcinogenesis is a prolonged and multi-phase process that involves initiation, promotion, progression, and metastasis as an outcome of an unbalanced cell proliferation and cell apoptosis /death [152]. Anti-proliferative agents are often associated with anti-mutagenic and hence anti-tumor activities.

The majority of the anticancer chemotherapeutics prescribed nowadays, such as alkylating agents, antibiotics, compounds targeting the cell cycle (microtubules, G1/S/G/M checkpoint proteins), and topoisomerase inhibitors, are cytotoxic compounds. These compounds are developed to kill carcinoma cells/tissues more effectively than normal cells/tissues because they generally target the more rapidly dividing tumor cells. However, this may bring about side effects on some actively dividing normal healthy cells, like bone marrow cells, hair follicles and gastrointestinal epithelial cells. Notwithstanding the problems associated with the application of cytotoxic drugs, bioassays testing cytotoxicity toward a panel of cancer cell types represent a reliable approach to screen natural products with anticancer potential, which has led to the successful discovery of anti-cancer pharmaceuticals like paclitaxel and camptothecin [153]. Table 1 summarizes the known fungal pigments with antitumor activities. These pigments are produced by fungi from a variety of species and ecological niches, including species in the genus *Monascus*, endophytic fungi, marine fungi, mushrooms, and fungi residing in special habitats. Together, they have demonstrated activity against a variety of cancer cell lines.

#### 5.2.2. Anti-Biofilm Activity

Melanin from the wood-ear edible fungus *Auricularia auricula* has been demonstrated to display distinct anti-biofilm effects towards *Escherichia coli* K-12, *Pseudomonas aeruginosa* PAO1, and *P. fluorescens* P-3, respectively, while no inhibitory activities were observed on bacterial growth [181]. Zhu et al. demonstrated that pigments from *A. auricula* can repress the production of violacein, a quorum-sensing signal for biofilm formation in the reporter strain *Chromobacterium violaceum* CV026 [182]. It is believed that the ability to form biofilm constitutes the predominant virulence factor for bacterial pathogens, as the biofilm formation is known to account for 80% of bacterial infections in humans [183]. Furthermore, biofilms represent a major cause of nosocomial infections, especially related to the emergence of multi-drug resistant strains, thereby contributing to refractory infectious diseases in humans. Thus, the quest of novel compounds that may effectively inhibit these biofilm-forming bacteria has been a hot topic in both pharmaceutical and clinical settings.

#### 5.2.3. Photosensitizers

Perylenequinones (PQs) represent a class of fungal pigments with a signature 3,10-dihydroxy-4,9-perylene-quinone chromophore. PQs are known as reactive oxygen species (ROS)-generating photosensitizers in medical and agricultural settings. Consequently, PQs have attracted significant attention [184]. Indeed, PQs-producing bambusicolous fungal species *Shiraia bambusicola* has long been used in Chinese folk medicine to treat stomach ache, rheumatic pain, as well as some dermatologic disorders like vitiligo and psoriasis [185]. Interestingly, PQs have also been extracted from fungal fruiting bodies, where a diverse microbial community typically resides. At present, how the PQs are produced in fungal fruiting bodies is not known [186].

Due to the complexity and difficulty of the chemical synthesis of PQs [187], mushroom fruiting bodies have been the major source for the supply of PQs. However, recent work by Ma et al. revealed that *Pseudomonas fulva* SB1 isolated from the fruiting body of the fungus *Shiraia bambusicola* was able to boost the production of fungal PQs, including hypocrellins A, C (HA and HC), and elsinochromes A–C (EA, EB and EC). The results revealed that following two days of co-cultures, *Shiraia* mycelial cultures exhibited the highest production of HA, about 3.2-fold of that in axenic culture. The co-culture might have led to the elicitation of fungal conidiation and the formation of compacted fungal pellets, as compared to axenic culture. Furthermore, the bacterial co-culture might have up-regulated the expression of polyketide synthase gene (PKS) and activated genes of the ATP-binding cassette (ABC) transporter as well as major facilitator superfamily transporter (MFS) for PQ exudation [186].

#### 5.2.4. Cholesterol-Lowering and/or Anti-Atherosclerotic Agents

In view of the association of obesity with an elevated risk of developing diabetes as well as cardiovascular disorders, the search for the cholesterol-lowering compounds has attracted much attention from chemists, biologists, pharmacists and medical practitioners. Monascin and ankaflavin, the two yellow pigments from *Monascus* spp., are found to have a remarkable antiobesity activity in a 3T3-L1 preadipocyte model of rat [71]. Their hypolipidemic effects were attributed to: (i) the inhibition of the differentiation and lipogenesis of preadipocytes by downregulating CCAT/enhancer-binding protein β (C/EBPβ) expression and its downstream peroxisome proliferator-activated receptor γ (PPARγ) and CCAT/enhancer-binding protein α (C/EBPα) expressions, and (ii) the inhibition of lipogenesis by increasing lipase activity and decreasing heparin releasable lipoprotein lipase (HR-LPL) activity [72]. Notably, the actions of monascin and ankaflavin do not resemble that of monacolin K (viz. lovastatin), a well-known cholesterol-lowering drug, which elevates creatine phosphokinase (CPK) activity, known to be a rhabdomyolysis marker [72].

Epidemiological studies have revealed a potential role of carotenoids in preventing cardiovascular disease (CVD) [8]. Reductions of low density lipoprotein (LDL) oxidation and of oxidative stress during plaque formation were thought to account for their effects. An association has been found between low serum lycopene level and an increased risk of atherosclerotic vascular events in middle-aged men [188]. Recent studies have demonstrated that fungal species such as *Blakesleea trispora* and *Rhodotorula* can produce lycopenes [189], thereby indicating their potential roles in the production of pharmaceuticals, food additives, or nutritional supplements that could be beneficial for patients with CVD.

#### 5.2.5. Promising Anti-Alzheimer Agents

As one of the most common forms of dementia, Alzheimer’s disease (AD) results in huge economic, emotional, and healthcare costs to individuals, families, and societies throughout the world. At present, there is a relative shortage of therapies available to treat AD. The aggregation of the microtubule-associated protein tau is suspected to be a seminal event in AD, thereby reducing/eliminating tau aggregation represents a potential therapeutic target for AD treatment and prevention. Paranjape et al. screened *Aspergillus nidulans* secondary metabolites for their capability to inhibit tau aggregation in vitro using an arachidonic acid polymerization protocol [190]. An aggregation inhibitor, asperbenzaldehyde, was identified. Asperbenzaldehyde is an intermediate in azaphilone biosynthesis. The authors further examined 11 azaphilone derivatives to determine their inhibitory activities against tau aggregation in vitro. All compounds examined were able to inhibit tau filament assembly to some degree, and four of the eleven compounds exhibited a pronounced activity of disassembling pre-formed tau aggregates in a dose-dependent fashion.

**Figure 12:**
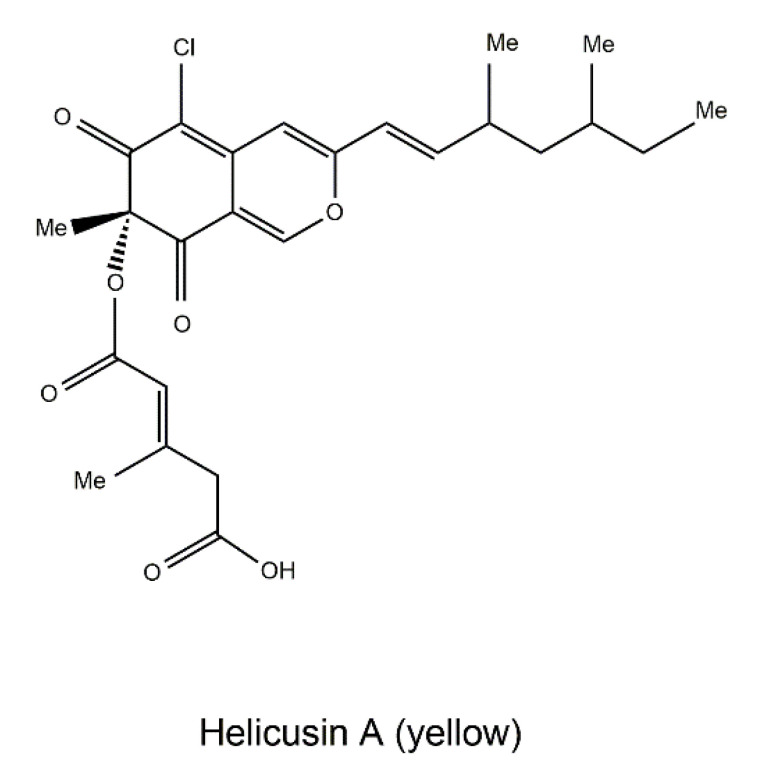
Structure of helicusin A, a pigment from marine fungus *Bartalinia robillardoides*.

Similarly, a detailed investigation into the metabolites of the Mediterranean sponge *Tethya aurantium*-associated fungus *Bartalinia robillardoides* strain LF550 led to the isolation and identification of new chloroazaphilones, helicusin E, isochromophilone X and isochromophilone XI, along with the known pigment helicusin A [191]. Based on the bioassays of these chloroazaphilones, helicusin A (Figure 12) was found to be a potent acetylcholinesterase inhibitor with an IC_50_ value of 2.1 μM. Given that inhibition of acetylcholinesterase is widely considered a therapeutic target for ameliorating AD [192], helicusin A is seen as a promising lead compound to develop a potential novel pharmaceutical regime against AD.

We should note that the anti-Alzheimer activities of the above-mentioned pigments have only come from in vitro studies. Such in vitro results are different from the fungal pigments already in clinical use, such as the cholesterol-lowering and/or anti-atherosclerotic agent monascin as described in 5.2.4. However, the preliminary results as demonstrated by fungal pigments are showing great promise as anti-Alzheimer’s disease drugs.

#### 5.2.6. Anti-Inflammatory Activity

Hsu et al. (2011) reported the isolation of an azaphilonidal derivative monaphilone A as well as ankaflavin from the fermented products of *Monascus purpureus* NTU 568 [193]. Further studies showed that ankaflavin and monaphilone A decreased lipopolysaccharide (LPS)-induced inflammatory responses including production of nitrite, and expressions of inducible nitric oxide synthase (iNOS) and cyclooxygenase 2 (COX-2) in murine macrophage RAW 264.7 cells, pinpointing the anti-inflammatory effects of these two fungal pigments.

#### 5.2.7. Antimicrobial Activities

A number of fungal pigments have shown significant antimicrobial activities [194,195]. Table 2 summaries the results from a few representative studies over the last decade. These pigments were from a variety of fungi living in diverse ecological niches, including marine environments, soil, and plants. The pigment-producing fungi are also broad, including both unicellular forms such as *Rhodotorula glutinis* and filamentous fungi such as *Monascus ruber* and species in the genera *Aspergillus, Fusarium,* and *Penicillium* (Table 2). Furthermore, most of these fungal pigments demonstrated broad activities against different groups of microorganisms, including plant and human fungal pathogens as well as both Gram-positive and Gram-negative bacterial pathogens. Of special note are pigments chaetoviridide A and B from the deep sea-derived filamentous fungus *Chaetomium* sp. NA-S0-R1 and pigments penicilones B−D from *Penicillium janthinellum* strain HK106 isolated from mangrove soil that showed strong activities against the methicillin-resistant *Staphylococcus aureus* (MRSA) [196,197].

#### 5.2.8. Others

Aside from the above-described roles of fungal pigments in human health, other human health related roles have also been reported. Those included antioxidative, cytotoxic, and immunosuppressive roles. Below are brief descriptions of those studies.

Anti-oxidative properties are commonly found among fungal pigments. The conjugated polyene chain in the carotenoids provides chemical reactivity against oxidizing compounds and free radicals that may otherwise result in damage to cellular functions. Apart from the anti-oxidative actions of carotenoids previously reviewed elsewhere [15], investigation of the Chinese medicinal fungus *Phellinus igniarius* has led to the isolation and identification of three fungal pigments derived from pyrano[4,3-c]isochromen-4-one, phelligridins H, I, and J [198]. These orange or yellow pigments all showed antioxidant activity, as evidenced by inhibiting rat liver microsomal lipid peroxidation with IC_50_ values of 4.8, 3.7, and 6.5µM, respectively, for phelligridins H, I, and J. In addition, phelligridins H and I inhibited protein tyrosine phosphatase 1B (PTP1B), while phelligridin J exhibited cytotoxic activity against four human cancer cell lines, A2708 (ovary cancer), A549 (lung cancer), Bel-7402 (hepatoma) and HCT-8 (colon cancer) [198]. As PTP1B is an important player in cell proliferation, differentiation, and malignancy, being involved, for instance, in the development of breast cancer, lung cancer, and esophageal squamous cell carcinoma [199,200]. The above findings highlight the potential of these novel pigments as antitumor agents.

The macula of human eye contains two carotenoids, lutein and zeaxanthin. Based on the NIH Eye Disease Case-Control Study [208], the dietary intake of antioxidants such as carotenoids is highly correlated to the incidence of age-related macular degeneration (AMD), demonstrating a statistically significant linear trend associating the reduction of risk with increasing consumption of carotenoids. It is widely recognized that these carotenoids may protect the macula from light-induced damage and scavenge free radicals generated in the photoreceptors [8].

Four new azaphilones, namely longirostrerones A−D, along with three known sterols, have been isolated from extracts of the Thai soil fungus *Chaetomium longirostre* [209]. Longirostrerones A−D exhibited potent cytotoxicity against KB (human epidermoid carcinoma of the mouth) cell lines with IC_50_ of 1.04, 1.52, 0.23, and 6.38 μM, respectively. Among these azaphilones under study, longirostrerone C showed significant cytotoxicity against KB (IC_50_ = 0.23 μM), close to the control drugs doxorubicine and ellipticine. Moreover, longirostrerones A showed strong inhibitory effects on human breast adenocarcinoma MCF7 and small-cell lung cancer NCI-H187 cancer cell lines with IC_50_ values of 0.24 and 3.08 μM, respectively. Apart from the antitumor activities shown above, longirostrerones A−C also displayed antimalarial activity against *Plasmodium falciparum* with the IC_50_ values ranging from 0.62 to 3.73 μM.

An early report revealed the pronounced immunosuppressive activities of monascin and ankaflavin, two yellow oligoketide pigments isolated from the mycelium of *Monascus purpureus* [210]. By inhibiting murine T-splenocyte proliferation, monascin and ankaflavin could interact with regulatory mechanisms of the immune system and thus suppress its function, indicating that the two *Monascus* pigments might serve as promising immunosuppressants.

## 6. Structure–Activity Relationship (SAR) Studies of Fungal Pigments

The structure–activity relationships have been investigated for several groups of fungal pigments. For example, the presence of a halogen atom at C-5, a proton at C-8 and a diene moiety in the C-3 side chain of the 6-oxoisochromane ring in azaphilones are necessary for gp120-CD_4_ binding, as exemplified by isochromophilones and their derivatives [211]. Furthermore, (an) electrophilic ketone(s) and/or enone(s) at both C-6 and C-8 in the 6-oxoisochromane ring are indispensable for the activity of cholesteryl ester transfer protein (CETP) inhibition, which is found in the azaphilone pigments such as chaetoviridin B, sclerotiorin, and rotiorin, etc. [78].

**Figure 13:**
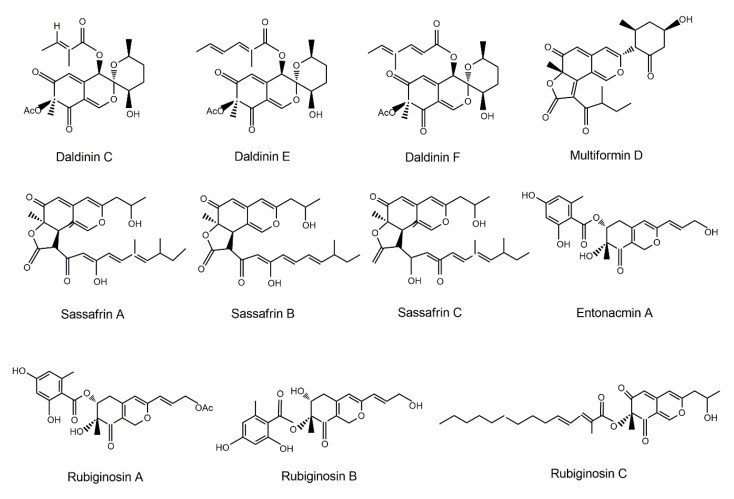

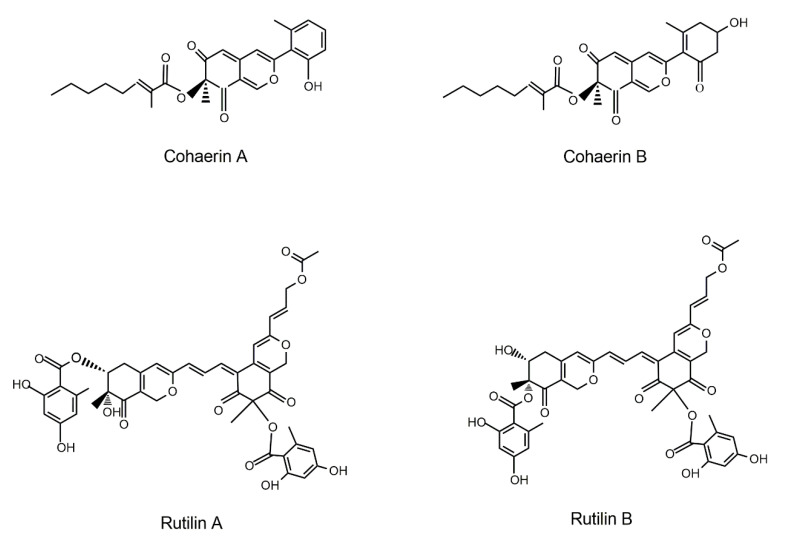
Structures of azaphilones.

In addition to the above-mentioned bioactivities, studies with murine macrophage cells RAW 264.7 have demonstrated that the inhibition of NO production relies on the structure of azaphilones [212]. For instance, spiro-derivatives daldinins C, E, and F (structures shown in Figure 13) only exert weak inhibition on NO production. In contrast, azaphilones bearing a lactone ring such as multiformin D and sassafrins A–C (Figure 13), are shown to exert stronger inhibitory actions on NO production than daldinins. Entonaemin A, rubiginosins A and B (Figure 13) are all azaphilones where an orsellinic acid moiety is attached to the bicyclic core via an ester linkage. However, since only rubiginosin A displays strong activity in this subclass of azaphilones, the location of the orsellinic acid moiety must not influence the activity of the compounds. Nonetheless, the presence of an acetyl group (such as in the case of rubiginosin A) is indispensable for an elevation of activity. Azaphilones with a fatty acid side chain linked to a bicyclic azaphilone core via an ester bond, like rubiginosin C, cohaerins A and B (Figure 13), demonstrated only weak activities. The most potent inhibitors of NO production seem to be dimeric azaphilones, such as rutilins A and B (Figure 13). Overall, while the acetyl group is necessary for inhibition, the location of orsellinic acid does not seem to change their activities [212]. Taken together, these studies showed that the inhibitory effects of azaphilones are substantially fortified by the number of orsellinic acid moieties in the molecule and the presence of conjugated double bonds in dimeric compounds [212].

Previous studies have shown that the antioxidant properties of the carotenoids are tightly correlated with their chemical structure. Both the polyene chain and these other structural features influence the chemical properties (e.g., redox properties) of the carotenoids [213]. For example, the structure–activity relationship was investigated by Rodrigues and his colleagues for fifteen carotenoids, showing that the opening of the β-ionone ring and the increase of chromophore extension in the carotenoid structure could be the predominant factors giving rise to the elevated capacity of peroxyl radical scavenging [214]. Specifically, the conjugated polyene chromophore gives rise to not only the light absorption properties, and thus hues, but also the chemical properties of the compound and hence light-harvesting and photoprotective activity. In addition, the polyene chain is the main determinant responsible for antioxidant role of carotenoids due to its chemical reactivity with free radicals, and oxidizing agents. Furthermore, carotenoids are found to be in precise locations and orientations within subcellular structures in vivo. Thus, their chemical and physical traits are strongly affected by other molecules in their vicinity (particularly proteins as well as membrane lipids), and vice versa [215]. Indeed, structural characteristics, such as size, shape, and polarity, are critical to the capability of a carotenoid to fit precisely into its molecular milieu to allow for its functionality. The role of carotenoids in influencing cell membrane-associated molecular processes by modifying the structure, properties, and stability of these membranes may constitute a significant aspect of their beneficial effects on human health.

Apart from their antioxidant actions, studies with marine carotenoids have unveiled the relationship between structure and anti-obesity activity. The key structure responsible for anti-obesity activity is thought to be the carotenoid end of the polyene chromophore bearing an allenic bond and two hydroxyl groups [216].

## 7. Conclusions and Further Prospects

Fungal pigments have primarily been known for their extensive application as colorants in the food, cosmetic and textiles industries for the past several decades. However, their applications other than as colorants are very broad, including as cholesterol-lowering drugs, exemplified by monacolin K, a clinically used drug also called lovastatin [217], and various anti-microbial and antitumor agents. Unexpected functions of new pigments as well as known ones are continuously being revealed. Fungal pigments have attracted significant interests from the pharmaceutical industry as sources of potential drugs to fight many life-threatening diseases such as cardiovascular disorders, Alzheimer’s disease, human carcinomas (hepatoma, breast, lung, colorectal, gastric, pancreatic, leukemia, hematopoietic, renal cell, and other cancers), infectious diseases, and parasitic diseases such as malaria. Since many fungal pigments have shown potential relevance to human health, there is increasing interest in understanding their mechanisms of action to pave the way for the future development of therapeutics against both acute and chronic diseases. Among the fungal pigments, melanin stands out for two reasons: (i) melanin confers excellent protection from such stressors as radiation, thermal fluctuations and drought, thereby giving an insight into the development of radiation-protective agents and devices for the human exploration into the outer space; (ii) melanin is known to contribute to pathogenesis and drug resistance in pathogenic fungi, and consequently represents an important target for the treatment of recalcitrant fungal infection in humans.

Thanks to advancements in microbiological, biochemical, genetic and genomics technologies and the development of high-throughput bioassay systems as well as the bioinformatics analyses, there are several potentially fruitful areas of research on fungal pigments. First, there is a diversity of fungal pigments produced by evolutionarily distant fungi. At present, the origins for the genes and the regulatory pathways governing their synthesis are largely unknown. Phylogenomic and transcriptomic studies should provide novel insights in this area. Second, a standardized test system and/or protocol should be established for screening the bioactivity of fungal pigments for both in vitro and in vivo assays, including appropriate positive and negative controls. Third, the structure–activity relationships are unknown for most fungal pigments. Finally, while the focus of our review is on colored fungal pigments, fungal metabolites that impact pigment productionunpub in both fungi and other organisms, including precursors and regulators for and metabolic products of pigments but they themselves are not colored, represent an additional source of fungal metabolites that have also been explored for human health benefits. One such metabolite in this category is kojic acid, a compound obtained from the filamentous fungus *Aspergillus oryzae* [218]. While colorless itself, kojic acid can inhibit tyrosinase which is essential for the synthesis of melanin in human skin and, consequently, it has been broadly used in dermatological applications [219]. With the increasing demand from the pharmaceutical industry, fungal pigments and their related products are expected to be an excellent resource for the design and development of novel therapeutic molecules in the future.

## Figures and Tables

**Table 1:** Fungal pigments with antitumor activities.

Fungal Sources	Fungal Species/Strain	Isolated Compound	Chemical Nature	Tumor Model/Cell Lines/Target Enzyme	Activity/Active Concentration	References
*Monascus*-fermented red rice	*Monascus pilosus*	Monascin & ankaflavin rubropunctamine & monascorubramine	Azaphilones	Ames test and Peroxynitrite-and UVB-induced mouse skin carcinogenesis model	Accelerate the mutagen decomposition	Ho et al. [154] Hsu et al. [155] Akihisa et al. [156]
	*Monascus* sp.	Ankaflavin		HepG2, A549 ^#^/IC_50_	15 μg/mL	Su et al. [157]
	*Monascus purpureus*	Monaphilone A Monoaphilone B		HEp-2, WiDr ^#^/IC_50_ HEp-2, WiDr/IC_50_	72.1, 55.8 μM 77.6, 55.3 μM	Hsu et al. [158]
		Rubropunctatin		BGC-823 ^#^/IC_50_ and in vivo mouse model	12.57 μM	Zheng et al. [159]
	*M. purpureus*	Monascopyridine C & D		IHKE (kidney epithelial cell) CCK8 assay/EC_50_	20.7–43.2 μmol/L	Knecht et al. [160]
	*Monascus* sp.	Glutamic acid derivative of *Monascus* orange pigments† (S)-(+)-1-amino-2-propanol derivative of the above orange pigments†		B16F10 (mouse melanoma cells) tyrosinase expression	30% inhibition 35% inhibition	Jo et al. [161]
	*Monascus pilosus*	Monascuspiloin	Monascin analog	PC-3 tumors of nude mice	42.5% inhibition (in vivo)	Chiu et al. [162]
Endophytic fungi	A fungus endophytic to *Mimosops elengi*	Ergoflavin	Xanthenes	ACHN (renal cell carcinoma), H460 (non-small-cell lung carcinoma), Panc1(pancreas), HCT116 (colon cancer), and Calu-1 (lung carcinoma)	1.2, 4.0, 2.4, 8.0, 1.5μM/IC_50_	Deshmukh et al. [163]
	*Phomopsis longicolla,* endophytic to *Dicerandra frutescens*	Dicerandrol A, Dicerandrol B, Dicerandrol C	Xanthenes	A549, HCT116 ^#^/IC_50_ A549, HCT116 A549, HCT116	7.0, 7.0 μg/mL 1.8, 1.8 μg/mL 1.8, 7.0 μg/mL	Wagenaar & Clardy [164]
	*Chaetomium globosum* endophytic to *Ginkgo biloba* *Penicillium* sp. CR1642D endophytic to Costa Rican rainforest	Chaetomugilides A–C	Azaphilone alkaloids	HepG-2^#^	1.7−3.4 μM/IC_50_	Li et al. [165]
		Penexanthone A Penexanthone B Dicerandrols B	Xanthones	A panel of cancer cell lines (Myeloma, lymphoma, leukemia, breast, prostate), also showing enhanced effects regarding tumor-stromal interaction	1−17 μM/IC_50_ IC_50_ of 1.2 μM (+stroma) *vs.* 2.4 μM (-stroma) in RPMI8226; 3.4 μM (+ stroma) *vs* 10.2 μM (-stroma) in H929	Cao et al. [166]
	*Chaetomium globosum* endophytic to marine fish *Mugil cephalus*	Chaetomugilin A Chaetomugilin C Chaetomugilin F	Azaphilone alkaloids	P388(murine), HL-60 (human) leukemia	8.7, 7.3 μM/IC_50_ 3.6, 2.7 μM/IC_50_ 3.3, 1.3 μM/IC_50_	Yasuhide et al. [167]
Marine fungi	*Aspergillus* *tubingensis* GX1-5E	TMC 256 A1	Naphtho-γ-pyrone	MCF-7 & MDA-MB-435 (breast carcinoma), Hep3B & Huh7 (hepatoma), SNB19 & U87 MG (glioblastoma)	19.92−47.98 μM/IC_50_	Sakurai et al. [168] Huang et al. [169]
	*Penicillium* *pinophilum* Hedgcok	Pinophilin A Pinophilin B	Hydrogenated azaphilones	Mammalian DNA polymerases (pols)A, B, Y	48.6–55.6 μM/IC_50_	Myobatake et al. [170]
	*Diaporthe* sp. SCSIO 41011	*epi*-isochromophilone II isochromophilone D	Chloroazaphilones	ACHN, 786-O, OS-RC-2 (three renal carcinoma) 786-O renal carcinoma	4.4, 3.0, 3.9 μM/IC_50_ 8.9 μM/IC50	Luo et al. [171]
	*Chaetomium* *globosum* HDN151398	*N*-glutarylchaetoviridin C Chaetomugilin A Chaetomugilin C	chloroazaphilones Azaphilone Alkaloids	MGC-803, HO8910 ^#^ HL-60, HCT-116 ^#^ HL-60, HO8910	6.6, 9.7 μM/IC50 6.4, 6.1 μM/IC50 6.6, 8.8 μM/IC50	Sun et al. [172]
	*Nigrospora* sp. strain 1403	Bostrycindeoxybostrycin	Anthraquinones	A549, HepG2 ^#^ A549, HepG2	2.64, 5.90 μg/mL 2.44, 4.41 μg/mL	Xia et al. [173]
	*Penicillium* sp.	(+)-formylanserinone B anserinones B	Pentaketides	MDA-MB-435 ^#^	2.90 μg/mL 3.60 μg/mL	Gautschi et al. [174]
Fungi in special habitats	*Pleurostomophora* sp. from a copper mine of North America	Berkchaetoazaphilones A, C Berkchaetorubramine berkchaetoazaphilone B	Azaphilones	Caspase-1 MMP-3 ^ξ^ Y79 ^#^ LOX IMVI ^#^	150,25,50 μM/IC_50_ 130,15, 45 μM/IC_50_ 1.1 μM /IC_50_ 10 μM/IC_50_	Stierle et al. [175]
	*Coniella fragariae* from goose dung	Coniellin A Coniellin A, D, E	Azaphilones	MDA-MB-231^#^	4.4 μM /IC50 and suppress tumor migration by 98% at 10 μM	Yu et al. [176]
Macrofungi (mushroom)	*Lactarius subvellereus*	Subvellerolactone B, Subvellerolactone D, Subvellerolactone E	Sesquiterpene hydroxylactones	A549, SK-MEL-2 ^#^, HCT-15 A549, HCT-15 A549, HCT-15	26.5, 18.3, 14.2 μM/IC50 25.1,17.8μM/IC50 19.6, 28.7μM/IC50	Kim et al. [177]
	*Boletus pseudocalopus*	Grifolin derivatives 1–3	Phenolic compounds	A549, B16F1 (mouse melanoma)	5.0–9.0 μg/mL 3.5–7.3 μg/mL	Song et al. [178]
	*Albatrellus confluens*	Albatrellin	Meroterpenoid	HepG2	1.55 μg/mL	Yang et al. [179]
	*Albatrellus flettii*	Grifolin, neogrifolin, confluentin	Phenolic compounds	SW480 & HT29 (two colon cancer lines)	35.4, 30.7μM/IC50 34.6, 30.1μM/IC50 33.5, 25.8μM/IC50	Yaqoob et al. [180]

^†^ rubropunctatin & monascorubin. ^ξ^ Matrix metalloproteinase-3 (MMP-3). ^#^ a variety of human cancer cell lines: A549 (lung adenocarcinoma), HepG2 (heptoblasoma), HEp-2 (laryngeal carcinoma), WiDr (colon adenocarcinoma), MGC-803 (gastric adenocarcinoma), HO8910 (ovarian cancer), HL-60 (promyelocytic leukemia), HCT-116 (colon cancer), MDA-MB-435 (breast cancer), Y79 (retinoblastoma), LOX IMVI (melanoma), SK-MEL-2 (skin melanoma), HCT-15 (colon adenocarcinoma).

**Table 2:** Antimicrobial activities of fungal pigments.

Fungal Sources	Fungal Species/Strain	Bioactive Component	Target Microbes ^1^	Antimicrobial Assay ^2^	Reference
Marine sponge-associated, Indonesia	*Trichoderma parareesei*	Yellow pigment	*Salmonella typhi, Escherichia coli,* multi-drug resistant strain	MIC: 1000 μg/mL (weak)	Sibero et al. [201]
Deep sea, West Pacific Ocean	*Chaetomium* sp. NA-S0-R1	Chaetoviridide A, B	*Vibrio rotiferianus*, *Vibrio vulnificus* and MRSA (*Staphylococcus aureus* ATCC 43300 & CGMCC 1.12409)	MIC: 7.3–7.8μg/mL	Wang et al. [197]
Spoiled onion	*Penicillium purpurogenum*	Red exopigment	*S. aureus, Salmonella typhi, E. coli, Corynebacterium diptheriae, Pseudomonas aeruginosa*	Agar diffusion assay showing inhibition zone (diameter 1.5–2.3 cm)	Patil et al. [202]
Tropical Culture Collection André Tosello (Campinas, SP, Brazil).	*Monascus ruber* CCT 3802	Orange pigments (monascorubrin, rubropunctatin) Red pigments (monascorubramine, rubropunctamine)	Foodborne bacterium *S. aureus* ATCC 25923, *S. aureus* ATCC 25923, *E. coli* ATCC 25922	Radial diffusion assay showing inhibition zone (diameter 0.15 cm) Inhibition zone (diameter 0.35, 0.63 cm, respectively)	Vendruscolo et al. [203]
Stressed environment	*Fusarium sp.*	Reddish orange pigment	*Klebsiella pnuemoniae, E. coli, Shigella sp.* (bacteria) *Aspergillus niger, Candida albicans* (fungi)	Well diffusion assay showing inhibition zone (diameter 1.6–2.9 cm)	Mani et al. [204]
Western Ghats forest, India	*Penicillium* sp. MF5	Yellow pigments	*Bacillus subtilis*	MIC: 12.5 μg/mL	Saravanan & Radhakrishnan [205]
Persian type culture collection (PTCC), Tehran, Iran	*Rhodotorula glutinis* PTCC 5256	Carotenoid pigments	*S. aureus*, *Bacillus cereus*, *Streptococcus pyogenes*, *E. coli*, *Salmonella enteritidis*, *Enterococcus faecalis*, *Listeria monocytogenes*	Disk diffusion assay showing inhibition zone (diameter 0.9–1.1 cm)	Yolmeh et al. [206]
Endophyte on marine brown algae, eastern China	*Aspergillus versicolor*	Asperversin, brevianamide M	*E. coli*, *S. aureus*	Disk diffusion assay showing inhibition zone (diameter 2.0–2.2 cm)	Miao et al. [207]
Endophyte on leaves of *Panax notoginseng*	*Emericella* sp. XL029	14-hydroxyltajixanthone 14-hydroxyltajixanthone, its hydrate, chloride derivative as well as epitajixanthone hydrate	Fungus- *Drechslera maydis, Rhizoctonia cerealis*, *Fusarium oxysporum* and *Physalospora piricola* Effective against all tested bacteria (except for drug resistant *Staphylococcus aureus*)	MIC: 25 μg/mL MIC: 12.5–50 μg/mL	Wu et al. [195]
Mangrove rhizosphere soil	*Penicillium janthinellum* HK1-6	Penicilones B−D	MRSA (*S. aureus* ATCC 43300, ATCC 33591)	MIC: 3.13–6.25 μg/mL	Chen et al. [196]

^1^, MRSA: Methicillin-resistant *Staphylococcus aureus*; ^2^, MIC: minimum inhibitory concentrations.
